# Identification and validation of ferroptosis-related biomarkers and the related pathogenesis in precancerous lesions of gastric cancer

**DOI:** 10.1038/s41598-023-43198-4

**Published:** 2023-09-26

**Authors:** Yuhui Kuang, Kuo Yang, Lingkai Meng, Yijia Mao, Fangbiao Xu, Huayi Liu

**Affiliations:** 1 Graduate School, Tianjin University of Traditional Chinese Medicine, Tianjin, 301608 China; 2 Department of Digestive Diseases, Tianjin Academy of Traditional Chinese Medicine Affiliated Hospital, Tianjin, 300120 China; 3 Graduate School, Henan University of Chinese Medicine, Zhengzhou, 450046 China

**Keywords:** Biological techniques, Immunology, Biomarkers, Diseases, Gastroenterology

## Abstract

Using advanced bioinformatics techniques, we conducted an analysis of ferroptosis-related genes (FRGs) in precancerous lesions of gastric cancer (PLGC). We also investigated their connection to immune cell infiltration and diagnostic value, ultimately identifying new molecular targets that could be used for PLGC patient treatment. The Gene Expression Omnibus (GEO) and FerrDb V2 databases were used to identify FRGs. These genes were analysed via ClueGO pathways and Gene Ontology (GO) enrichment analysis, as well as single-cell dataset GSE134520 analysis. A machine learning model was applied to identify hub genes associated with ferroptosis in PLGC patients. Receiver Operating Characteristics (ROC) curve analysis was conducted to verify the diagnostic efficacy of these genes, and a PLGC diagnosis model nomogram was established based on hub genes. R software was utilized to conduct functional, pathway, gene set enrichment analysis (GSEA) and gene set variation analysis (GSVA) on the identified diagnostic genes. Hub gene expression levels and survival times in gastric cancer were analysed using online databases to determine the prognostic value of these genes. MCPcounter and single-sample gene set enrichment analysis (ssGSEA) algorithms were used to investigate the correlation between hub genes and immune cells. Finally, noncoding RNA regulatory mechanisms and transcription factor regulatory networks for hub genes were mapped using multiple databases. Eventually, we identified 23 ferroptosis-related genes in PLGC. Enrichment analyses showed that ferroptosis-related genes were closely associated with iron uptake and transport and ferroptosis in the development of PLGC. After differential analysis using machine learning algorithms, we identified four hub genes in PLGC patients, including MYB, CYB5R1, LIFR and DPP4. Consequently, we established a ferroptosis diagnosis model nomogram. GSVA and GSEA mutual verification analysis helped uncover potential regulatory mechanisms of hub genes. MCPcounter and ssGSEA analysed immune infiltration in the disease and indicated that B cells and parainflammation played an important role in disease progression. Finally, we constructed noncoding RNA regulatory networks and transcription factor regulatory networks. Our study identified ferroptosis-related diagnostic genes and therapeutic targets for PLGC, providing novel insights and a theoretical foundation for research into the molecular mechanisms, clinical diagnosis, and treatment of this disease.

## Introduction

Precancerous lesions of gastric cancer (PLGC) refer to the early stages of gastric cancer. The widely recognized worldwide progression model for gastric cancer is “normal gastric mucosa—chronic superficial gastritis—chronic atrophic gastritis—intestinal metaplasia—dysplasia—gastric cancer”. This series of stages, known commonly as the Correa cascade reaction, manifests in the gastric mucosa before the initiation of gastric cancer^1^. Early identification of these precancerous lesions can reduce the incidence of gastric cancer. Gastric cancer (GC) is one of the most common cancers in the world, ranking fourth in incidence and third in mortality among malignant tumours^2^. Despite various treatment methods, including surgery, radiotherapy and chemotherapy, targeted therapy, immunotherapy, and traditional Chinese medicine, the treatment effect for gastric cancer remains limited.

Screening for GC precancerous lesions primarily relies on gastroscopy, an invasive and costly procedure that decreases its widespread use. There are also no current serum biomarkers for diagnosing PLGC. Recently, efforts to discover novel biological indicators for PLGC diagnosis and investigate PLGC’s immune cell infiltration components have increased, potentially revolutionizing the screening, diagnosis, and treatment of PLGC and mitigating the advancement and initiation of gastric cancer.

Ferroptosis is a regulated cell death mechanism first introduced in 2012 that is primarily induced by iron-mediated oxidative damage, lipid peroxidation, and cellular membrane injury^3^. In addition to its strong implications in the proliferation of numerous diseases, it has recently become an important area of interest in cancer research^4^. Emerging studies indicate that ferroptosis significantly participates in the progression and onset of gastrointestinal diseases such as inflammatory bowel disease^5^, gastric cancer^6^, and colorectal cancer^7^. Interestingly, specific genes linked to ferroptosis exhibit potential as biomarkers for predicting gastric cancer^8^.

**Figure 1:**
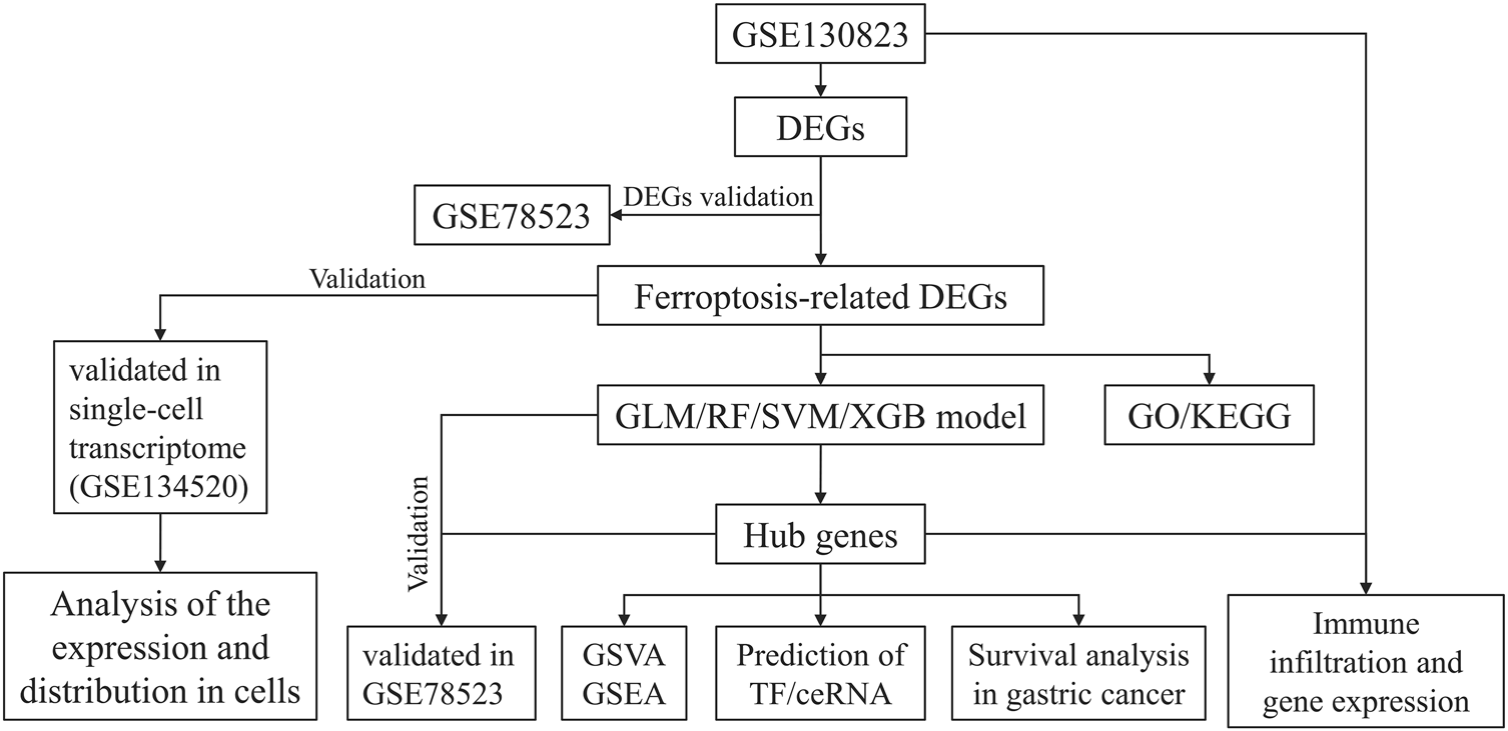
Flow chart illustrating the study design and methodology.

Currently, there is no research investigating the molecular mechanisms linking ferroptosis and PLGC. Therefore, this study aims to analyse existing datasets related to PLGC to identify diagnostic genes and therapeutic targets associated with ferroptosis and PLGC. Our analysis will focus on key genes and their potential pathways, enabling us to conduct preliminary research into their effects and provide new insights for clinical and basic research into PLGC. A roadmap of our research approach is illustrated (Fig. 1).

## Materials and methods

### Data source and normalization

This study utilized the keywords “precancerous lesion of gastric cancer” for retrieval and screening purposes, ultimately acquiring two bulk RNA-seq datasets (GSE78523 and GSE130823) related to PLGC. Specifically, the GSE130823 dataset contained 31 PLGC tumour samples and 47 gastritis controls, while the GSE78523 dataset included 14 PLGC samples and 15 healthy control samples after excluding intestinal epithelial samples that did not progress to cancer during follow-up. All the relevant datasets used in this study were retrieved from the Gene Expression Omnibus (GEO) database (https://www.ncbi.nlm.nih.gov/geo/). The FerrDb V2 database was searched^9^ (http://www.zhounan.org/ferrdb) to obtain the ferroptosis-related gene set. The raw count matrix was log2-corrected using the normalizeBetweenArrays function of the limma^10^ package.

### Identification of differentially expressed genes and ferroptosis-related genes

This study employed the R package Limma^10^ to conduct differential analysis on each of the two datasets. Probe sets that lacked corresponding gene symbols were removed, and genes with multiple probe sets were averaged. Genes exhibiting a *P* value < 0.05 and |fold change|≥ 1 were identified as differentially expressed genes. The Venn diagram between the two datasets and the ferroptosis-related gene set was analysed and drawn using the R package VennDiagram^11^, with intersecting genes defined as key genes related to ferroptosis. Using data from the GSE130823 dataset, Spearman^12^ correlation analysis was performed on the ferroptosis-related genes. Heatmaps were subsequently plotted to illustrate the expression levels of the ferroptosis-related gene set for samples from both datasets.

### Expression of ferroptosis-related gene sets in different cells of PLGC

To further validate the relevance of ferroptosis in PLGC progression, this study utilized the UCell^13^ package to analyse the expression of the ferroptosis-related gene set in various cells from patient tissues with intestinal epithelial dysplasia taken from the GSE134520 single-cell dataset. The GSE134520 dataset comprised four patients with severe intestinal epithelial dysplasia. Batch effects were removed using harmony, and tSNE dimensionality reduction and singleR cell annotation were used before analysing the expression of the ferroptosis-related gene set in different cells and plotting an expression profile^14^.

### Enrichment analyses of ferroptosis-related genes

In this study, we used the R package clusterProfiler to conduct Gene Ontology (GO) analysis on ferroptosis-related genes, as well as ClueGO for pathway enrichment analysis from the KEGG database^15^. The GO database provides comprehensive gene annotation information, while the ClueGO-derived pathway analysis helps us understand the biological processes, signalling pathways, and other functions that these genes may activate. This approach is an effective means of analysing the potential molecular mechanisms underlying these genes.

### Identification of ferroptosis-related hub genes by machine learning

This study utilized four different machine learning models, including random forests^16^ (RF), support vector machines^17^ (SVM), eXtreme Gradient Boosting^18^ (XGBoost), and generalized linear models^19^ (GLM), to help screen for core genes within the ferroptosis-related gene set. By identifying the top 10 shared features from each of the four models, we identified hub genes related to ferroptosis in PLGC through intersection analysis of these identified genes.

### Diagnostic value and validation of hub genes

To further explore the diagnostic value of hub genes, this study analysed and validated their diagnostic efficacy in two datasets using the R package pROC^20^. For clinical convenience, a logistic regression model was constructed to build a PLGC diagnostic model based on ferroptosis-related hub genes, and a nomogram was drawn. The reliability of the model was verified by decision curve analysis (DCA) and the C-index.

### Gene set enrichment analysis (GSEA) and gene set variationt analysis (GSVA) of hub genes

To more accurately explore the differences in signalling pathway activation caused by differential expression of hub genes in diseases, this study divided the samples into two groups based on the median expression level of hub genes: high expression and low expression. Using the signalling pathway dataset from the MSigDB database^21^ (https://www.gsea-msigdb.org/gsea/msigdb) as background, GSEA and GSVA enrichment analyses were conducted. Both GSEA and GSVA algorithms are based on gene expression levels and calculate the differences in pathway activation between the two groups. To ensure accuracy, this study used both GSEA and GSVA algorithms to mutually verify the results.

### Survival analysis

We conducted an analysis of the correlation between the expression levels of hub genes in gastric cancer and survival time using the Kaplan‒Meier Plotter online survival analysis website (http://kmplot.com/analysis/). This helped us understand the prognostic value of analysing hub genes.

### Immune infiltration analysis

To further analyse the immune microenvironment of PLGC patients' lesion tissue, this study used the GSE130823 dataset to analyse the infiltration of immune cells through the MCPcounter^22^ algorithm, analysed the infiltration and immune function of immune cells through single-sample gene set enrichment analysis (ssGSEA), analysed the differences in immune cells between the PLGC and control groups through the limma^10^ package, and analysed the correlation between hub genes and immune cells through the Spearman^12^ method.

### LncRNA-miRNA and transcription factor (TF) prediction of hub genes

To further investigate the regulatory patterns of hub genes, this study screened for miRNAs that could regulate hub genes by using three online databases, miRTarBase^23^ (https://mirtarbase.cuhk.edu.cn), Starbase^24^ (https://starbase.sysu.edu.cn/starbase2), and TargetScan^25^ (https://www.targetscan.org). Then, by using the spongeScan^26^ database (https://spongescan.rc.ufl.edu/), lncRNAs that could regulate these miRNAs were identified. The TF prediction was based on the Enrichr database^27^ (https://maayanlab.cloud/Enrichr/) and involved selecting human transcription factors with a *P* value less than 0.05. Finally, the information was imported into Cytoscape software to generate a network regulatory diagram.

## Results

### Identification of differentially expressed genes and ferroptosis-related genes

**Figure 2:**
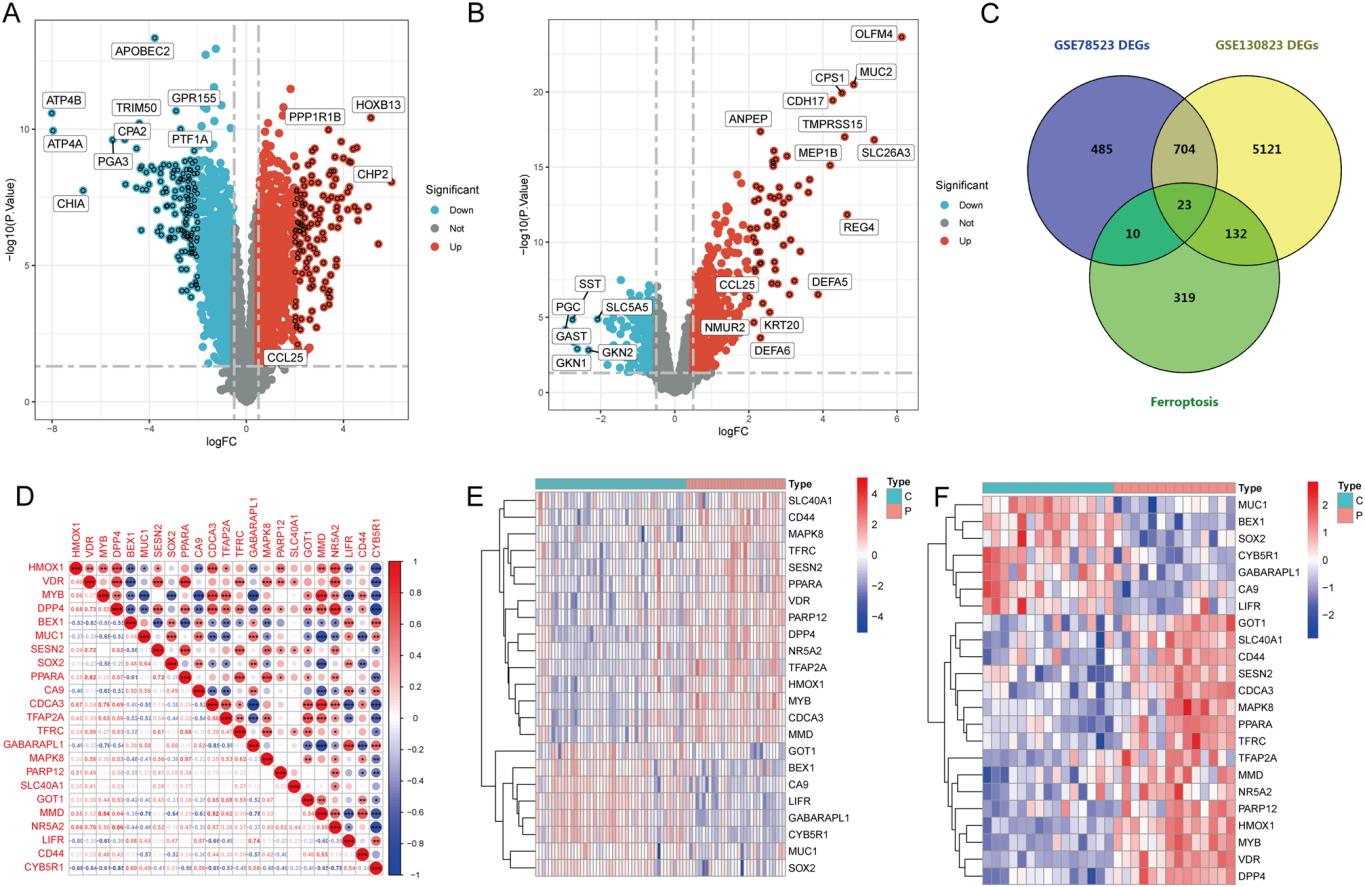
Differential analysis and identification of ferroptosis-related differentially expressed genes. (**A**) Volcano plot displaying differential gene expression in GSE130823. (**B**) Volcano plot illustrating differential gene expression in GSE78523. (**C**) Venn diagram showcasing the overlap between differentially expressed genes and ferroptosis-related genes in both datasets. (**D**) Correlation heatmap revealing the interplay among the differentially expressed ferroptosis-related genes. (**E**) Heatmap delineating the expression pattern of the differentially expressed ferroptosis-related genes in GSE130823. (**F**) Heatmap illustrating the expression pattern of the differentially expressed ferroptosis-related genes in GSE78523.

In this study, we conducted differential analysis on two chip datasets, GSE78523 and GSE130823, by sorting and analysing the data. The resulting differential volcano plots can be seen (Fig. 2A GSE130823, Fig. 2B GSE78523). A total of 1222 differentially expressed genes were identified in the GSE78523 dataset, while 5980 differentially expressed genes were identified in the GSE130823 dataset. Relevant gene information related to ferroptosis drivers, markers, and other parameters was collected from the FerrDb V2 database, and a total of 484 genes related to ferroptosis were identified. The intersection of the three datasets yielded 23 ferroptosis-related genes (Fig. 2C), which were used as a set in PLGC. We plotted heatmaps to better understand the expression of the ferroptosis-related gene set in each dataset (Fig. 2E GSE130823, Fig. 2F GSE78523). Next, we analysed the correlation between ferroptosis-related genes and created a correlation matrix heatmap (Fig. 2D). As demonstrated by the data, most genes showed a high degree of correlation with one another, indicating that the genes were closely related and that the gene set had some consistency in function, which is worth further investigation.

### Analysis of ferroptosis-related genes in single-cell RNA-seq

**Figure 3:**
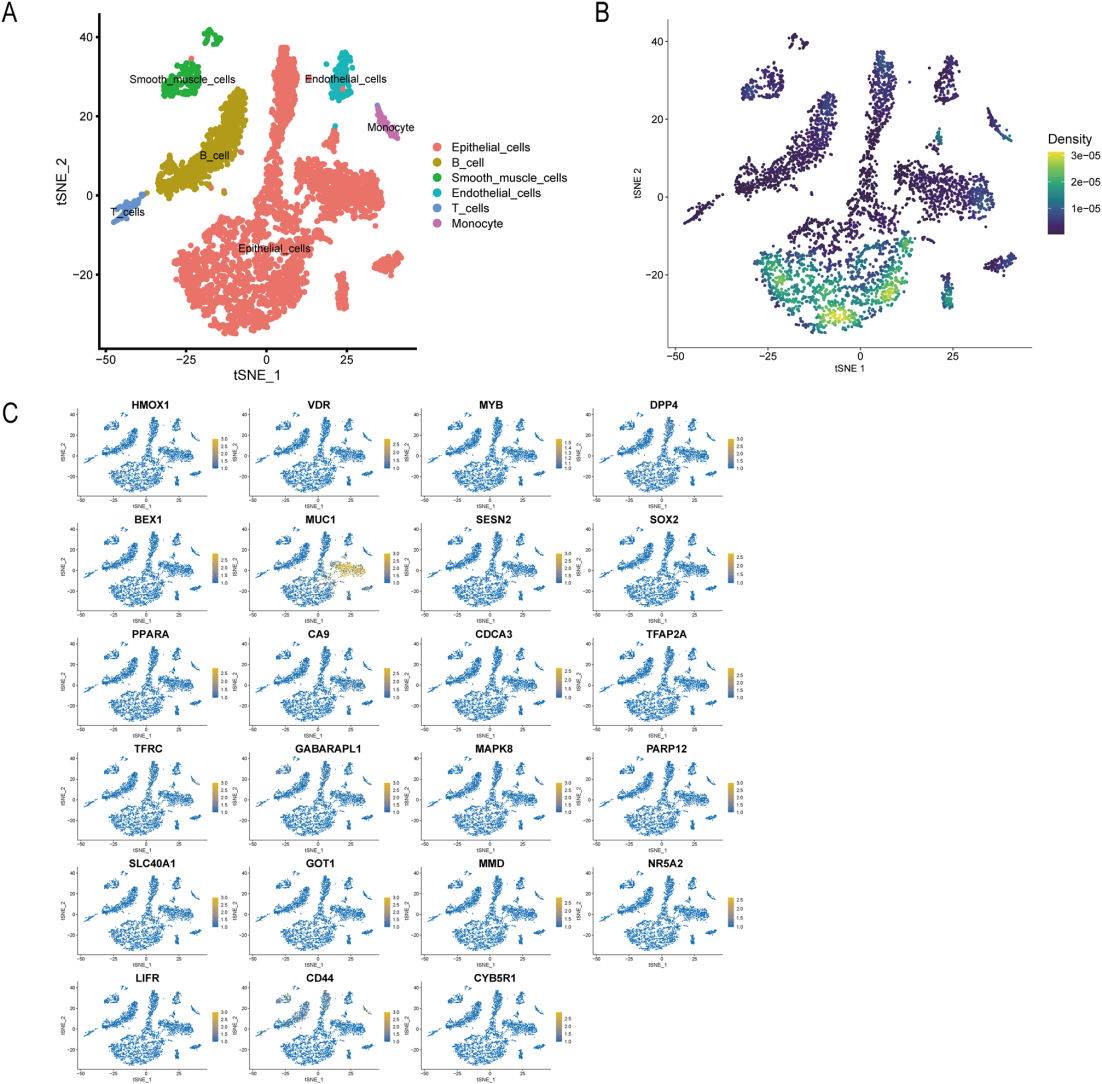
The investigation of ferroptosis-related genes within the single-cell dataset GSE134520 has been executed. (**A**) The cartography of individual cells is annotated. (**B**) The density of gene expression distribution pertaining to the ferroptosis-related gene set exhibited in the cellular population. (**C**) An examination of the distribution densities of each specific ferroptosis-related gene within constituent cells is undertaken.

To gain further insights into the expression patterns of the ferroptosis-related gene sets in PLGC intestinal epithelial transformation, a series of single-cell analysis techniques were employed to process the dataset and generate expression profiles for the ferroptosis-related gene sets across various cells (Fig. 3). Following singleR cell annotation, it was observed that the samples of intestinal epithelial transformation consisted of a relatively higher proportion of epithelial cells, which is consistent with the pathological progression of intestinal epithelial transformation in PLGC (Fig. 3A). Remarkably, the expression of the ferroptosis gene set was exclusively prominent in epithelial cells, indicating that ferroptosis plays a crucial role in the process of intestinal epithelial transformation (Fig. 3B). To provide further insights into the expression patterns of individual genes across different cells, we produced single-gene single-cell expression profiles (Fig. 3C). These profiles revealed significant expression levels of MUC1 and DPP4 in epithelial cells, which warrants further investigation.

### Enrichment analyses of ferroptosis-related genes

**Figure 4:**
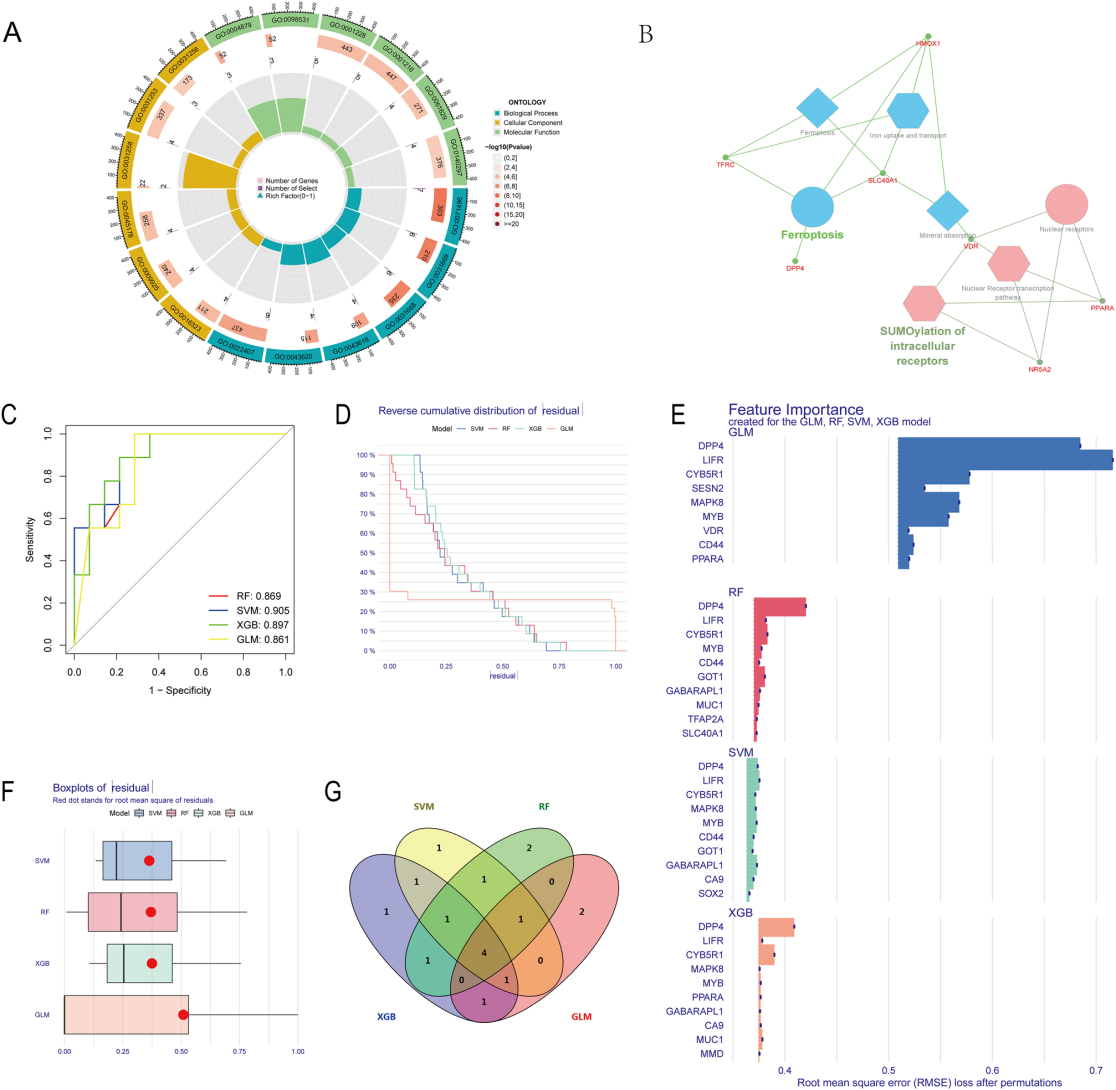
Enrichment analysis of ferroptosis-related differentially expressed genes in GO and KEGG pathways, as well as identification of hub genes. (**A**) GO enrichment analysis of ferroptosis-related differentially expressed genes. (**B**) KEGG enrichment analysis of ferroptosis-related differentially expressed genes. (**C**) ROC curves of all four machine learning models. (**D**) Residual distribution plots of machine learning models. (**E**) Histogram of feature contribution degrees of machine learning models. (**F**) Boxplots of residual values of machine learning models. (**G**) Venn diagram illustrating the overlap of features among all four machine learning models.

This study revealed enrichment of the PLGC ferroptosis-related gene set in signalling pathways and biological processes such as iron uptake and transport, mineral absorption, ferroptosis, and SUMOylation of intracellular receptors (Fig. 4A,B).

### Identification of ferroptosis-related hub genes

**Figure 5:**
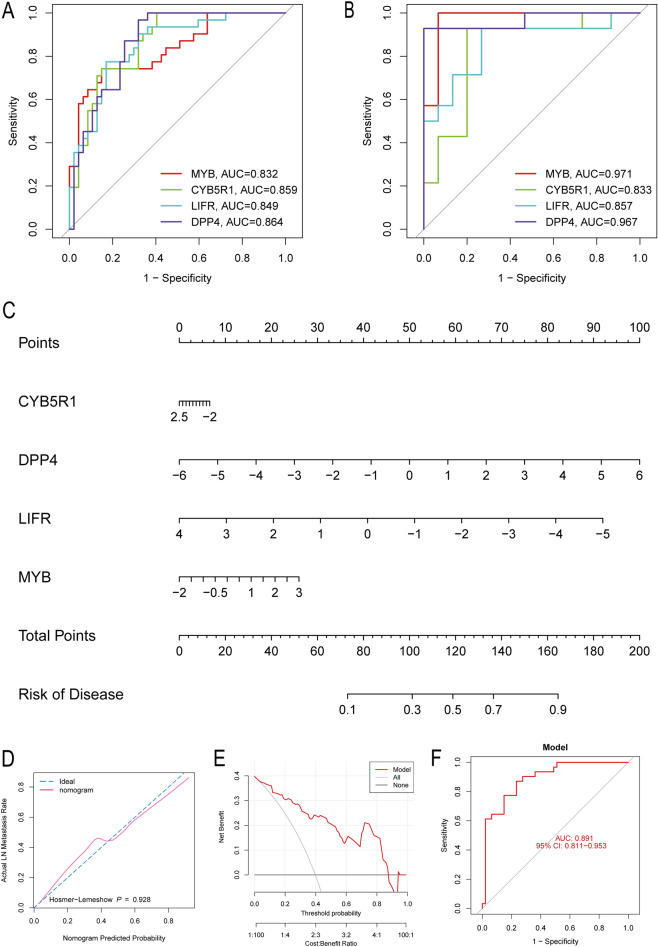
Hub genes and construction of the diagnostic model. (**A**) ROC curves of hub genes in GSE134520. (**B**) ROC curves of hub genes in GSE78523. (**C**) Nomogram of the diagnostic model. (**D**) C-index plot of the model. (**E**) DCA curve of the model. (**F**) ROC curves of the model.

We employed four different machine-learning algorithms to assist in selecting core genes within the ferroptosis gene set. ROC curves were used to evaluate the performance of these four models. As shown in Fig. 4C, each model achieved high diagnostic efficiency; RF had an AUC value of 0.869, SVM had 0.905, XGB had 0.897, and GLM had 0.861. We also assessed the stability of the models by plotting the residual distribution graph and residual boxplot, as shown in Fig. 4D and F, respectively. These graphs demonstrate that all four models are stable and have practical value. We then extracted the top ten contributing genes from each model and took their intersection (Fig. 4E,G), ultimately identifying four core genes: MYB, CYB5R1, LIFR, and DPP4. Finally, we evaluated the diagnostic efficacy of the core genes across multiple datasets (Fig. 5A GSE130823, Fig. 5B GSE78523). The AUC values of the four hub genes were consistently above 0.83 in both datasets, indicating their high diagnostic efficiency and confirming that using machine learning to select core genes is a reliable approach.

### Diagnostic value and validation of hub genes

To further investigate the diagnostic value of the hub genes and facilitate their clinical application, we created a column chart (Fig. 5C). We also used the Hosmer‒Lemeshow test to evaluate the accuracy of our model (Fig. 5D), achieving a P value of 0.928, which indicates that our model is highly accurate. Additionally, we plotted the decision curve analysis (DCA) curve (Fig. 5E), which revealed a high net benefit gain and demonstrated the potential for good clinical application. Furthermore, we plotted the ROC curve (Fig. 5F), which revealed an AUC value of 0.891. This indicates that our model has a high accuracy rate of 0.891 when diagnosing PLGC and provides reliable diagnostic results.

### Gene set enrichment analysis (GSEA) and gene set variation analysis (GSVA) of hub genes

**Figure 6:**
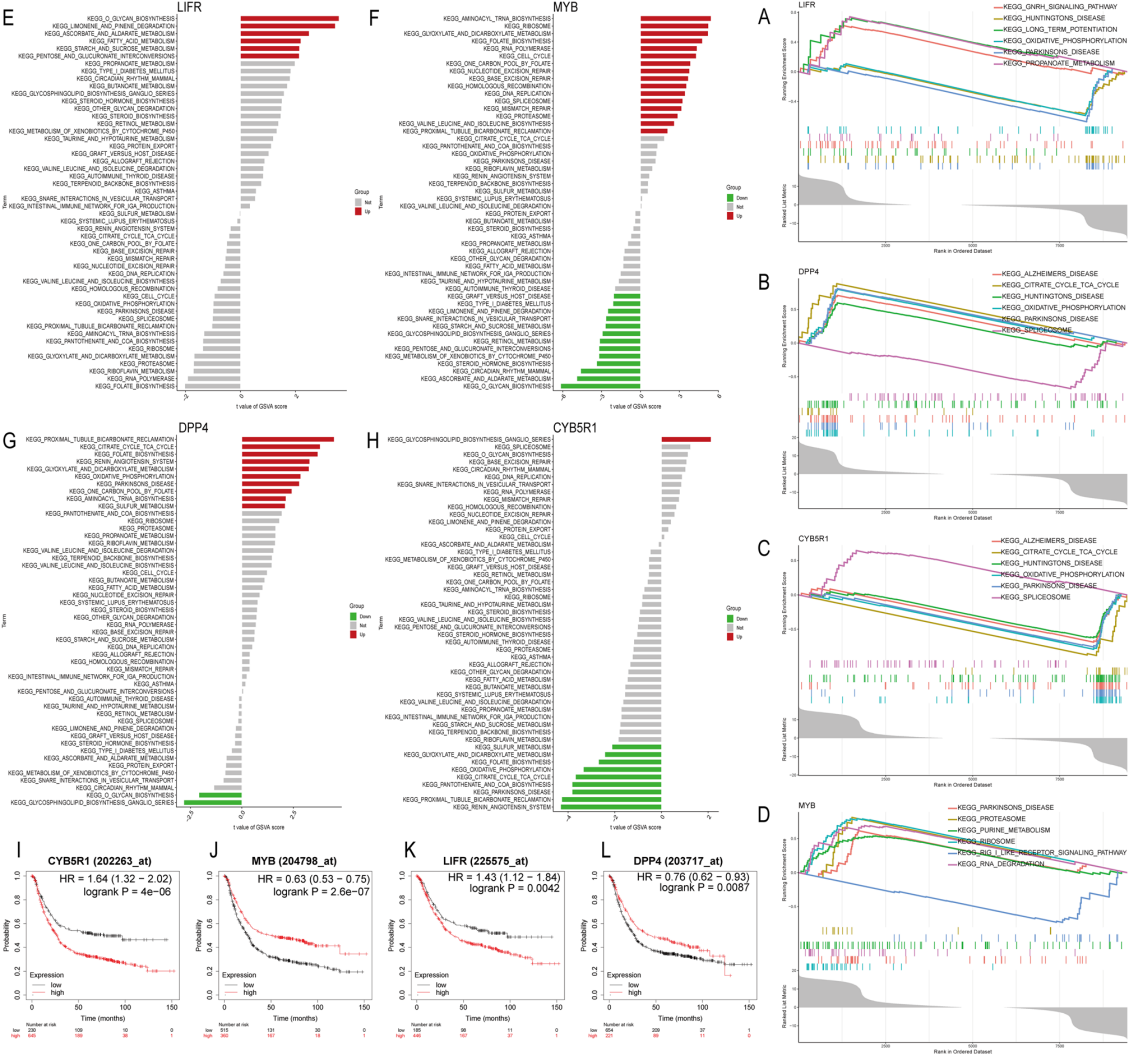
GSVA analysis, GSEA analysis, and survival analysis of hub genes. (**A**-**D**) GSEA analysis of hub genes. (**E**–**H**) GSVA analysis of hub genes. (**I**-**L**) Survival analysis of hub genes.

To further investigate the regulatory role of hub genes in diseases, we utilized two algorithms, GSEA and GSVA, to analyse and cross-validate the pathways activated by hub genes (Fig. 6). Based on the results, we found that CYB5R1 is downregulated, while both the oxidative phosphorylation and citrate cycle TCA cycle signalling pathways are activated. Interestingly, we also discovered that these signalling pathways are activated in the high DPP4 expression group, along with other disease signalling pathways, such as Parkinson's disease. Regarding LIFR GSEA (Fig. 6A), we found that the oxidative phosphorylation pathway was activated in the downregulated group; however, no significant differences were observed in the GSVA analysis (Fig. 6E). We also found that the O-glycan biosynthesis pathway and some sugar and fat metabolism signalling pathways, such as fatty acid and starch and sucrose metabolism, showed significant differences in GSVA analysis and were activated in the LIFR high-expression group.

Many pathways were activated by high or low MYB expression (Fig. 6D GSEA, 6F GSVA). Notably, we observed that the proteasome and ribosome signalling pathways were activated in the MYB high-expression group, while some sugar and fat metabolism signalling pathways, such as starch and sucrose metabolism and O-glycan biosynthesis, were activated in the MYB downregulated group. Furthermore, it is interesting to note that the O-glycan biosynthesis signalling pathway was reflected in the GSVA of all four hub genes.

### Survival analysis

We performed survival analysis on the correlation between hub gene expression levels and patient survival using the Kaplan‒Meier Plotter, a user-friendly and efficient online tool based on cancer-related datasets from the GEO database. Our analysis revealed that low expression levels of the CYB5R1 and LIFR genes were significantly associated with longer patient survival times, while high expression levels of the DPP4 and MYB genes were significantly associated with longer patient survival times (Fig. 6I–L).

### Immune infiltration analysis

**Figure 7:**
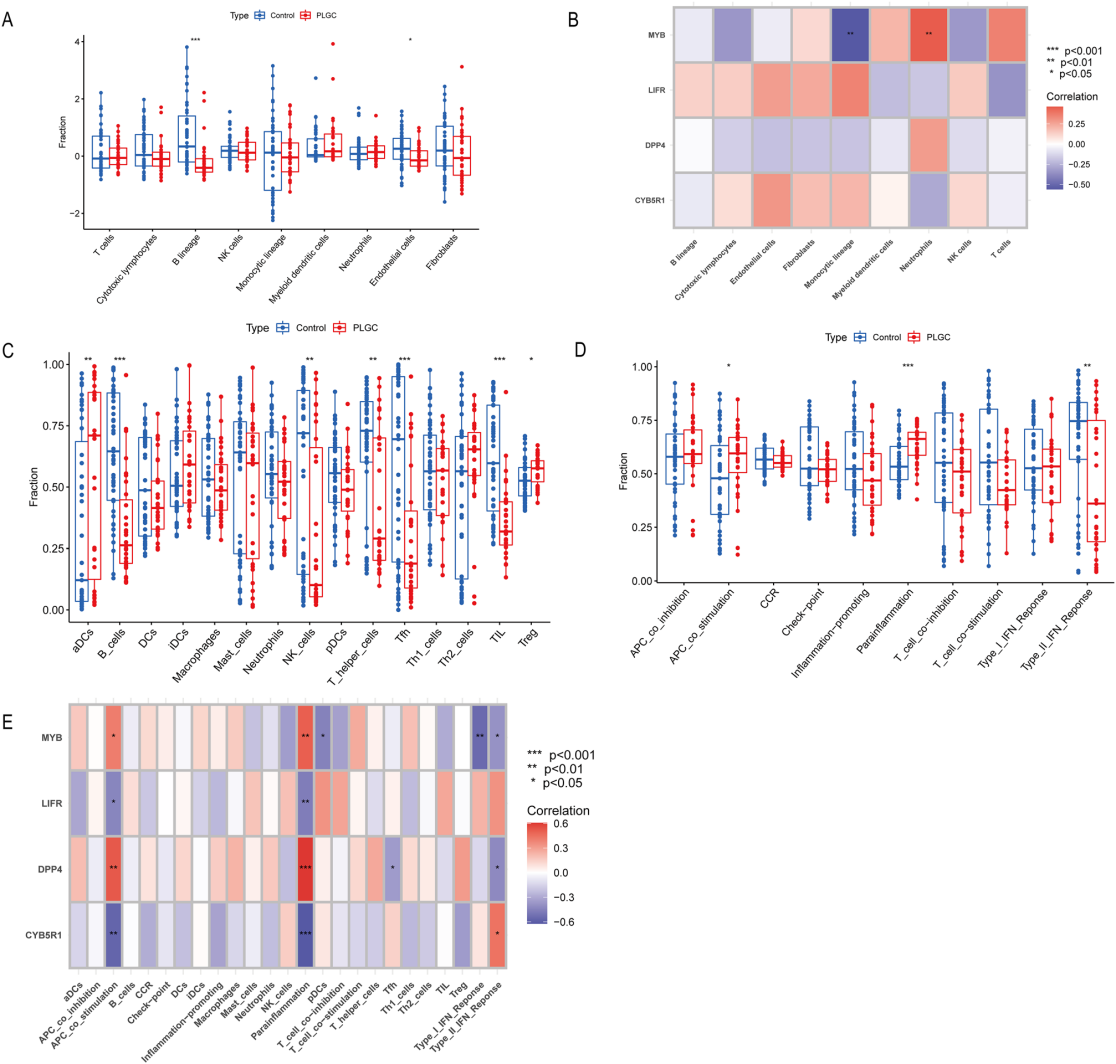
Immunological analysis. (**A**) MCPcounter immunological analysis. (**B**) Correlation analysis between hub genes and immune cells using MCPcounter. (**C**) ssGSEA analysis of immune cells. (**D**) ssGSEA analysis of immune functions. (**E**) Correlation analysis between hub genes and ssGSEA immune-related parameters.

To provide an accurate description of immune infiltration in PLGC, we used two algorithms—MCPcounter and ssGSEA—to calculate immune infiltration and function (Fig. 7A,C,D). The MCPcounter algorithm revealed significant decreases in B lineage and endothelial cells in the PLGC group. Meanwhile, the ssGSEA results indicated significant increases in activated dendritic cells (aDCs), B cells, NK cells, T helper cells, T follicular helper cells (Tfhs), and tumour-infiltrating lymphocytes (TILs) in the PLGC group. Furthermore, the immune function scoring results demonstrated significant increases in APC costimulation and parainflammation, while the type II IFN response was significantly decreased in the PLGC group. During the correlation analysis (Fig. 7B,E), we found a significant and positive correlation between APC costimulation and parainflammation with two genes, MYB and DPP4, both of which are associated with good prognosis when highly expressed. A significant and negative correlation was observed between APC costimulation and parainflammation with LIFR and CYB5R1, both of which are associated with poor prognosis when highly expressed. Additionally, MYB and DPP4 showed a significant negative correlation with the type II IFN response.

### ceRNA prediction and transcription factor of hub genes

**Figure 8:**
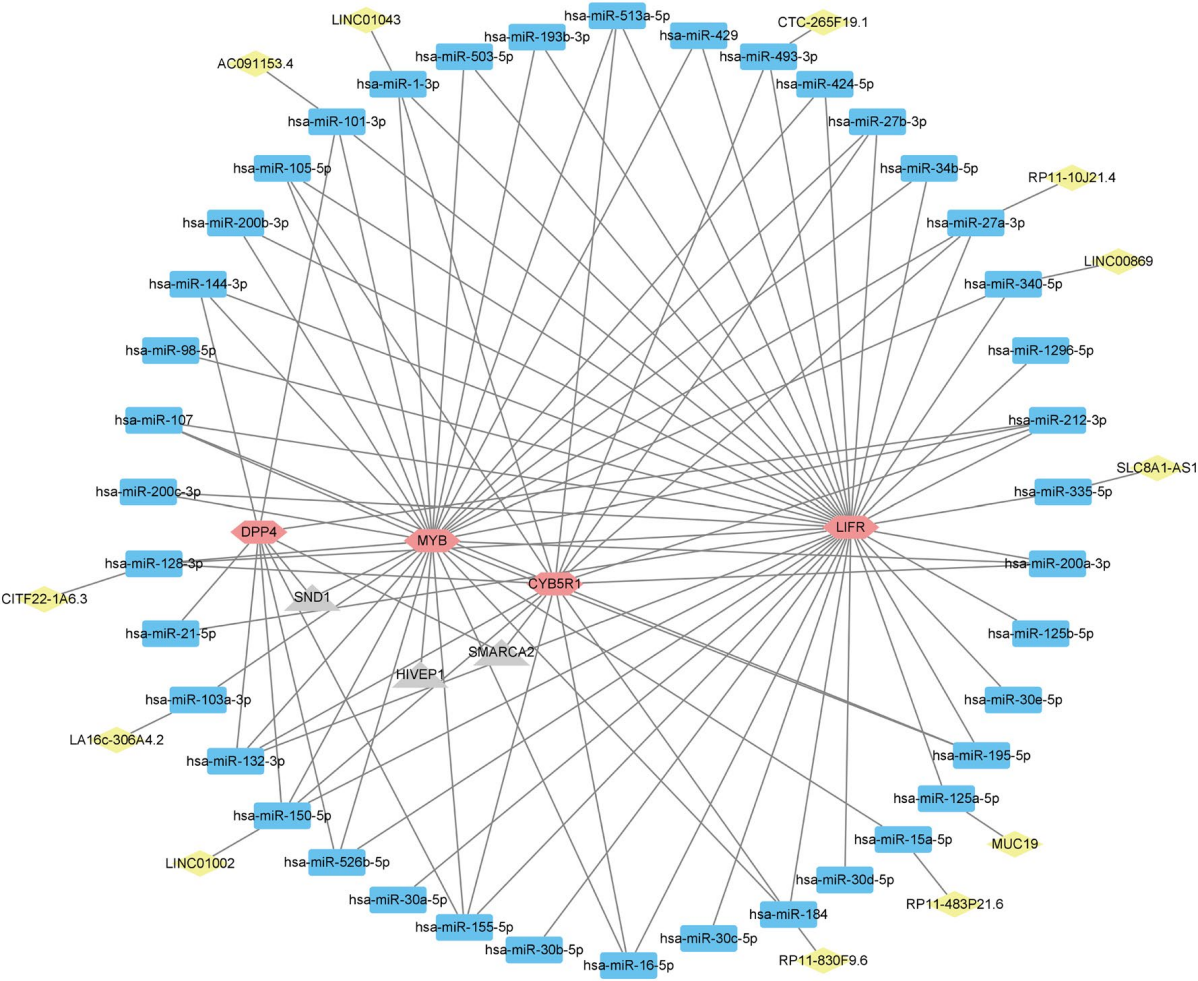
Regulatory network of lncRNA–miRNA–mRNA.

To further explore the noncoding RNA regulatory mechanisms and transcription factor regulatory networks of hub genes, we searched multiple online databases and constructed a network graph to display their regulatory properties (Fig. 8). In total, we identified three transcription factors, 40 miRNAs, and 11 lncRNAs. Among them, hsa-miR-150-5p was found to be associated with four hub genes and was regulated by LINC01002.

## Discussion

Gastric cancer, a prevalent malignant tumour type, primarily originates from preneoplastic lesions such as chronic atrophic gastritis, intestinal metaplasia, dysplasia, and carcinoma in situ. Notably, the appearance of atrophic gastritis and intestinal epithelial metaplasia mark critical stages in the progression towards gastric cancer. Therefore, to inhibit gastric cancer development, early detection and intervention of these precursor lesions are of pivotal importance.

Although iron is an indispensable nutrient for the human body, its overaccumulation can induce oxidative stress and tissue damage, which in turn are associated with various diseases, including cancer. Existing research points towards an increased risk of gastric cancer in relation to excessive iron, possibly driving the inflammation of gastric mucosa and leading to the accumulation and destruction of reactive oxygen species (ROS) in cellular components^28^. The relationship between iron-induced programmed cell death (ferroptosis) and precursor lesions of gastric cancer has been probed. The regulation of ferroptosis might thus be a promising preventive and therapeutic strategy for such precursor lesions, and this study potentially elucidates the mechanism that links ferroptosis with gastric precancerous lesions.

This paper utilized a comprehensive database search and in-depth analysis approach to identify 23 possible ferroptosis-related genes linked to PLGC. The identified genes were further validated within a single-cell dataset. A diagnostic model was then constructed using four machine learning methods, leading to the identification of MYB, CYB5R1, LIFR, and DPP4 as the prominent genes, each with an AUC value exceeding 0.83. The study consistently found that DPP4 ranked high in feature contribution analyses, further confirming its importance in the diagnostic model. Based on the nomogram, the expression levels of DPP4 and LIFR directly affected disease diagnosis, where patients with high DPP4 expression were more likely to have the disease, while LIFR had the opposite effect. Evaluation of the nomogram model using the C-index, DCA curve, and ROC curve demonstrated its effectiveness in diagnosing diseases. Moreover, variations in immune cell infiltration were evaluated using immunological methods such as ssGSEA and MCPcounter, revealing a significant decrease in the B-cell population within the PLGC group. This is concurrent with research suggesting the recruitment of B cells in providing an early response to *Helicobacter pylori* (*H. pylori*) infection by combating infection within the gastric mucosa^29^. However, disproportionate immune reactions may exacerbate chronic inflammation, fostering a cascade of reactions, inducing DNA impairment, and ultimately escalating cancer progression. As the inflammation becomes chronic, B-cell infiltration decreases and eventually becomes subdued; simultaneously, T cells and NK cells also show a significant reduction in their dissemination, indicating an urgent need to alleviate chronic inflammation, failing which the number of immune cells will eventually decrease, engendering a weakened immune response and establishing a conducive environment for carcinogenesis. Parainflammation within the tumour microenvironment contrasted with our studies, as we noted a heightened level of parainflammation within the PLGC group. This variation corresponded positively with MYB and DPP4; however, it showed an inverse relationship with LIFR and CYB5R1. This implies that parainflammation could play a pivotal role in the progression of PLGC, possibly affecting the onset and development of the disease in conjunction with other pathophysiological occurrences. Its function as an adaptive response of the immune system to minor tissue stress, transitioning between the basal state and an inflamed state, is well documented. Parainflammation aims to restore tissue function and homeostasis under normal conditions^30^. However, it could also act as a risk factor, inducing p53 mutations and driving cancer progression^31^. Meanwhile, several studies have demonstrated that parainflammation may accelerate ageing, promote atherosclerosis formation, and have other harmful effects^32^. Based on the results of this study, it is speculated that in PLGC, parainflammation may have exceeded the required amount for its physiological function and engendered a state of coexistence between physiological and pathological changes. Currently, no fundamental research validates the association between parainflammation and PLGC. To summarize, there needs to be a balance in parainflammation levels; any excessive levels could actively induce pathological transformations, while inadequate levels could contribute to a loss of physiological functions. Therefore, this issue demands more extensive research in the field.

Research indicates that CYB5R1 (NADH-cytochrome b5 reductase 1) synergizes with POR (cytochrome P450 oxidoreductase) to enhance the synthesis of harmful PLOOH (phospholipid hydroperoxide), thereby inducing cell ferroptosis^33^. Our research employed GSVA and GSEA analyses, which suggest that decreased levels of CYB5R1 trigger the oxidative phosphorylation and citrate cycle TCA signalling pathways. Increasing research attention is currently focused on cancer energy metabolism. Studies suggest that the tumour microenvironment is capable of modifying the energy metabolism of tumour cells, shifting from glucose oxidative phosphorylation and mitochondrial respiration to the use of aerobic glycolysis as the primary energy mechanism. Despite this, tumour cells thrive, continuously amassing energy for proliferation, a process known as the Warburg effect^34^. Parallel to our findings, lower CYB5R1 expression in gastric cancer is associated with extended survival rates by fostering normal cellular energy metabolism and decelerating the transformation process from intestinal metaplasia to cancer. Our findings suggest that CYB5R1 could be a potential therapeutic target to impede ferroptosis and delay the progression of PLGC. Furthermore, research on brain glioma has shown regulation of CYB5R1 through demethylation drugs such as 5'-aza-2'-deoxycytidine (aza-dC)^35^ introduces new perspectives for the treatment of PLGC.

Research suggests that DPP4 (dipeptidyl peptidase 4) may incite lipid peroxidation, thereby accelerating ferroptosis^36^. DPP4 has also been observed to suppress GLP1 (glucagon-like peptide 1) activity, resulting in increased blood glucose levels^37^. Furthermore, another study identified GLP1 as a significant trigger for cancer precursor lesions^38^. Our research indicates that DPP4 exhibits high expression in the PLGC group, and thus, it merits further exploration to ascertain whether this influence is exerted through GLP1 inhibition, a potential risk factor for cancer precursor lesions, or by fostering ferroptosis in cancerous cells to delay cancer progression. This investigation found that oxidative phosphorylation was also heightened in the DPP4 high-expression group, contradicting the energy metabolism mechanism of CYB5R1. Although there exists no direct research probing the correlation between the oxidative phosphorylation signalling pathway and DPP4, studies confirm that elevated DPP4 expression can suppress GLP1, thereby augmenting blood glucose levels. This phenomenon might stimulate the activation of the oxidative phosphorylation signalling pathway, enhancing the energy supply in the tumour microenvironment and potentially delaying cancer progression. This hypothesis requires additional research and verification, not only in the context of cancer precursor lesions but also in gastric cancer, given that high DPP4 expression has been associated with a significant improvement in patient survival time.

The proto-oncogene Myb (MYB), known for its role in promoting the progression of various types of cancer, is currently under investigation as a potential therapeutic target^39^. Despite the lack of studies that explore the relationship between MYB and gastric cancer or PLGC, our research has demonstrated its application in therapy. We found that high MYB expression in gastric cancer patients had an inverse relationship with their survival rate, adding a new twist to its established role as a proto-oncogene. The proteasome and ribosome signalling pathways, activated by high MYB expression, may influence this outcome.

Moreover, research indicates a decrease in the expression of the leukaemia inhibitory factor receptor (LIFR) in liver cancer, which leads to the promotion of ferroptosis and inhibition of tumour progression^40^. Concomitantly, an increase in LIFR expression has been linked to longer patient survival^41^. However, in our study, high LIFR expression corresponded with a shorter patient survival time, indicating the risky assumption of generalizing liver cancer results. LIFR, acting as a receptor subunit for leukaemia inhibitor factor (LIF), is said to boost gastric cancer cell proliferation, migration, and invasion through the LIFR-Hippo-YAP pathway^42^. It should be noted that LIF is functionless when LIFR expression is silenced. Although some studies have proposed that both LIF and LIFR can hinder tumour progression^43^, Guan et al.^44^ showed that lidocaine impairs gastric cancer development by upregulating LIFR. Our findings associate high LIFR expression with poor prognosis in gastric cancer patients. An O-glycan biosynthesis signalling pathway surfaced as the most significantly activated pathway in the high-expression group based on GSVA analysis, possibly promoting cancer precursor lesion progression as a downstream pathway of LIFR activation. To summarize, our results shed light on the complex role of LIFR in various types of cancer, indicating that its function is likely type dependent. Hence, there is an imminent need for more in-depth studies to acquire comprehensive knowledge of LIFR-mediated tumour progression, opening doors to new therapeutic targets.

Research has demonstrated that LIFR (leukaemia inhibitory factor receptor) expression is reduced in liver cancer, promoting ferroptosis and suppressing tumour progression^40^. Furthermore, elevated LIFR expression in liver cancer has been associated with prolonged patient survival^41^. However, in our study, high LIFR expression corresponded with a shorter patient survival time, indicating the risky assumption of generalizing liver cancer results. LIFR, acting as a receptor subunit for LIF (leukaemia inhibitory factor), is said to boost gastric cancer cell proliferation, migration, and invasion through the LIFR-Hippo-YAP pathway^42^. It should be noted that LIF is functionless when LIFR expression is silenced. Although some studies have proposed that both LIF and LIFR can hinder tumour progression^43^, Guan et al.^44^ showed that lidocaine impairs gastric cancer development by upregulating LIFR. Our findings associate high LIFR expression with poor prognosis in gastric cancer patients. An O-glycan biosynthesis signalling pathway surfaced as the most significantly activated pathway in the high-expression group based on GSVA analysis, possibly promoting cancer precursor lesion progression as a downstream pathway of LIFR activation. To summarize, our results shed light on the complex role of LIFR in various types of cancer, indicating that its function is likely type dependent. Hence, there is an imminent need for more in-depth studies to acquire comprehensive knowledge of LIFR-mediated tumour progression, opening doors to new therapeutic targets.

O-glycan biosynthesis demonstrated significant divergences among low-expression groups for DPP4 and MYB genes in the GSVA analysis. Karasawa et al.^45^ revealed that α1,4-linked N-acetylglucosamine residues (αGlcNAc) present in O-glycans can hinder the growth of gastric cancer by counteracting Helicobacter pylori infection and curtailing subsequent inflammation, which in turn exacerbates tumour development. This observation substantiates the crucial role of the O-glycan biosynthesis pathway in initiating carcinogenesis-related inflammation and correlates with the expression of LIFR, DPP4, and MYB.

Conclusively, this study used a spectrum of bioinformatics strategies to discover four central biomarkers linked to ferroptosis. Their potential mechanisms in disease genesis and progression were explored through a literature review. Moreover, probable regulators of noncoding RNAs and transcription factors were identified, which led to the development of a diagnostic model suitable for clinical application. These insights provide a fresh understanding of the drastic changes occurring with ferroptosis that contribute to the progression of PLGC.

## Data Availability

The datasets presented in this study can be found in online repositories. The names of the repository/repositories and accession number(s) can be found in the article. GSE130823 (https://www.ncbi.nlm.nih.gov/geo/query/acc.cgi?acc=GSE130823), GSE78523 (https://www.ncbi.nlm.nih.gov/geo/query/acc.cgi?acc=GSE78523) and GSE134520 (https://www.ncbi.nlm.nih.gov/geo/query/acc.cgi?acc=GSE134520).
